# Complete Genome Sequence of Human Oral Phylogroup 1 *Treponema* sp. Strain OMZ 804 (ATCC 700766), Originally Isolated from Periodontitis Dental Plaque

**DOI:** 10.1128/MRA.00532-20

**Published:** 2020-05-28

**Authors:** Yuki Chan, Yong-Biao Huo, Xiaolin Yu, Huihui Zeng, Wai Keung Leung, Rory M. Watt

**Affiliations:** a Faculty of Dentistry, The University of Hong Kong, Hong Kong SAR, China; University of Rochester School of Medicine and Dentistry

## Abstract

Host-associated treponeme bacteria play etiological roles in human and animal soft tissue infections. *Treponema* sp. strain OMZ 804 (ATCC 700766) was isolated from dental plaque sampled from a patient with periodontitis in Switzerland in 1994. We report here the complete genome sequence of its 2.98-Mb circular chromosome.

## ANNOUNCEMENT

The host-associated treponeme bacteria Treponema medium and Treponema vincentii play etiological roles in oral infectious diseases in humans and animals, especially periodontal diseases, and in various animal soft tissue infections (1–5). There are many uncertainties in treponeme taxonomy, with *T. medium*/*T. vincentii*-like taxa frequently identified within infected human and animal niches (4–9). *Treponema* sp. strain OMZ 804 (MH1F1, ATCC 700766) was originally isolated from the oral cavity of a periodontitis patient in Switzerland in 1994 (10). A previous multilocus sequence analysis (MLSA) study placed *Treponema* sp. strain OMZ 804 within a distinct species-level phylotype (*Treponema* sp. strain IC) that was phylogenetically related to *T. medium* and *T. vincentii* (9). Here, we report the complete genome sequence of *Treponema* sp. strain OMZ 804, obtained directly from the depositor, Chris Wyss.

Axenic culture of OMZ 804 was maintained anaerobically at 37°C in supplemented tryptone-yeast extract-gelatin-volatile fatty acids-serum (TYGVS) medium as described previously (9). Genomic DNA was extracted using a QIAamp DNA mini extraction kit (Qiagen, Germany) following the manufacturer’s protocol.

Long-read sequencing was performed on an Oxford Nanopore Technologies (ONT) minION Mk1B device with an R9.4 flow cell (FLO-MIN106). The whole-genome sequencing library was prepared using the ONT 1D genomic DNA ligation sequencing kit (SQK-LSK108) and barcoding kit (EXP-NBD103) according to the manufacturer’s protocol (version NBE_9006_v103_revP_21Dec2016), excluding the optional genomic DNA fragmentation step. The DNA was repaired using the NEBNext formalin-fixed, paraffin-embedded (FFPE) repair mix (New England Biolabs), cleaned with AMPure XP beads (Beckman Coulter), and dA tailed using the NEBNext end repair/dA-tailing module; then, sequencing adapters were ligated onto the prepared ends. ONT reads were base called with Albacore v2.3.3 (11), and adapters were removed from the sequence reads using Porechop v0.2.3 (12). The ONT sequencing generated a total of 13,606 reads that consisted of 169,920,109 bases, and the *N*_50_ value was 24,668.

Short-read sequencing was performed on the Illumina HiSeq 2500 platform at the Centre for PanorOmic Sciences (CPOS; The University of Hong Kong). Shotgun libraries for 101-bp paired-end reads (PE100) were prepared using the TruSeq DNA sample prep kit according to the manufacturer’s protocol (part number 15026486 Rev.C). The Illumina sequences were quality filtered to remove reads containing >5% of unknown bases, >50% of bases with a quality value of ≤10, and bases with a read length of <20 bp. Adaptor sequences were trimmed using Trimmomatic v0.39 (13). This yielded 5,974,798 reads and 603,431,232 bases (99.91%) after quality filtering.

Hybrid genome assembly was performed using Canu v1.8 (minReadlength = 5000) (14), with sequence polishing using Racon v1.3.1 (15) and Pilon v1.22 (16) correction using the Illumina reads. The completeness and circularity of the genome assembly were confirmed through mapping the Illumina and ONT reads using GraphMap v0.5.2 (17), placing the *dnaA* gene at the beginning of the genome assembly. Default parameters were used for all software unless otherwise specified. This resulted in one circular complete sequence (2.98 Mb; coverage, 260×) with a GC content of 44.3%.

The genome was annotated using Prokka v1.3 (18). A total of 2,891 protein-coding genes (CDSs), 49 tRNAs, and 6 rRNAs were annotated in the chromosome. Genome similarity calculated using orthologous average nucleotide identity (OrthoANI) (19) showed 84.7% identity to *T. vincentii* ATCC 35580 and F0403, 89.8% to *T. medium* ATCC 700293, and 90.0% to *Treponema* sp. strain OMZ 838 (20). The presence of PTK2 focal adhesion kinase 1 (EC 2.7.10.2), bacitracin transport system ATP-binding protein BcrA, and NADP-reducing hydrogenase subunit HndC (EC 1.12.1.3) are unique to this strain in comparison to other genome-sequenced phylogroup 1 treponeme species/phylotypes.

### Data availability.

The complete genome sequence has been deposited in GenBank under the accession number CP048020. The raw sequencing reads were deposited in the NCBI Sequence Read Archive (SRA) under the accession numbers SRR10954731 and SRR10954730 and BioProject accession number PRJNA602574.
